# Is ICI-based therapy better than chemotherapy for metastatic NSCLC patients who develop EGFR-TKI resistance? A real-world investigation

**DOI:** 10.3389/fonc.2022.920047

**Published:** 2022-08-23

**Authors:** Yajie Cheng, Bin Yang, Wen Ouyang, Chen Jie, Wei Zhang, Gang Chen, Junhong Zhang, Jing Yu, Conghua Xie

**Affiliations:** ^1^ Department of Radiation and Medical Oncology, Zhongnan Hospital of Wuhan University, Wuhan, China; ^2^ Hubei Key Laboratory of Tumor Biological Behaviors, Zhongnan Hospital of Wuhan University, Wuhan, China; ^3^ Hubei Cancer Clinical Study Center, Zhongnan Hospital of Wuhan University, Wuhan, China; ^4^ Hubei Cancer Hospital, Tongji Medical College, Huazhong University of Science and Technology, Wuhan, China

**Keywords:** EGFR-TKI resistance, EGFR-sensitive mutations, combined therapy, metastatic NSCLC, immunotherapy

## Abstract

**Purpose:**

To evaluate the outcomes of immune checkpoint inhibitor (ICI)-based treatments versus classical chemotherapy for metastatic non-small cell lung cancer (NSCLC) patients who develop epidermal growth factor receptor tyrosine kinase inhibitor (EGFR-TKI) resistance and to explore the population that may benefit from ICI-based therapy.

**Materials and methods:**

All patients who had previously received EGFR-TKI therapy at two cancer centers in China and developed resistance to targeted therapies were included. Progression-free survival (PFS) and overall survival (OS) were utilized to evaluate the outcomes of the study cohort.

**Results:**

A total of 132 patients were included. The median follow-up time for this cohort was 21.7 months (IQR, 14.8–28.8 months), calculated from the date of EGFR-TKI resistance. The median PFS and OS were 4.9 months (IQR, 2.8–9.2) and 13.5 months (IQR, 6.6–26.5 months), respectively. Multivariate analysis showed that ICI-based therapy could significantly improve OS when compared to the classic chemotherapy (hazard ratio [HR], 0.55; 95% CI, 0.34–0.88; *P* = 0.01) after adjusting for variables such as gender, age, mutation status, and brain or liver metastasis status. The combined modality of ICI plus chemotherapy could offer a long-term OS benefit in most subgroups, such as young (<65 years) patients, and those without secondary T790M mutations or absence of liver and brain metastases, and the populations with good Eastern Cooperative Oncology Group (ECOG) scores.

**Conclusion:**

For patients presenting with EGFR-TKI resistance, ICI-based therapy could offer a more favorable survival than classical chemotherapy. The combination of ICI with chemotherapy may be the optimal modality for those with good ECOG PS scores.

## Introduction

Lung cancer is currently the most prevalent malignancy worldwide (1). In recent years, with the introduction of immune checkpoint inhibitors (ICIs), such as programmed cell death-1 (PD-1) and programmed cell death-ligand 1 (PD-L1) antibodies, the outcomes of metastatic non-small cell lung cancer (NSCLC) have greatly improved (2–4). However, the responses to immunotherapy seem to differ according to differences in the inherent immune microenvironment (5, 6). For example, NSCLC patients without epidermal growth factor receptor (EGFR) or anaplastic lymphoma kinase (ALK) genetic aberrations (EGFR^−^/ALK^−^) seem to benefit from immunotherapy, while the response to immunotherapy seems to be poor in those who harbor EGFR-sensitive mutations and ALK rearrangements (EGFR^+^/ALK^+^) (7).

The tumor immune microenvironment (TME) may undergo changes as the disease progresses (8, 9). For example, one study found that NSCLC patients who developed resistance to first-generation EGFR tyrosine kinase inhibitors (TKIs) but did not have a secondary T790M mutation might benefit from ICI monotherapy due to an increase in PD-L1 expression and tumor mutation burden (10). Despite the benefits achieved, the results of ICI monotherapy after EGFR-TKI resistance were not yet satisfactory (11, 12).

Recently, a phase II study confirmed that ICI plus chemotherapy could be a promising second-line option for NSCLC patients developing EGFR-TKI resistance but without a secondary T790M mutation (13). However, a subgroup analysis of the IMpower150 showed that the combination of chemotherapy, bevacizumab, and ICI could only improve PFS but did not achieve an OS benefit when compared to bevacizumab plus chemotherapy (14). Therefore, we conducted this investigation of ICI-based therapy versus classic chemotherapy for those that developed EGFR-TKI resistance from two cancer centers in China and to explore the optimal treatment modality.

## Materials and methods

### Study cohort

All metastatic NSCLC patients (n = 110), either squamous or adenocarcinoma, who had previously benefited from EGFR-TKI, including first- and third-generation drugs, and have developed resistance at the Department of Radiation and Medical Oncology, Zhongnan Hospital of Wuhan University, were included in this study. They did not receive chemotherapy before or during treatment with EGFR-TKI. The diagnostic criteria of EGFR-TKI resistance were based on radiological or pathological results. In order to match the number of patients who underwent immunotherapy and chemotherapy alone, we additionally included a subset of patients (n = 22) who underwent immunotherapy after EGFR-TKI resistance occurring at the Department of Hubei Cancer Hospital, between September 2018 and July 2020.

This retrospective study was approved by the Department of Radiation and Medical Oncology, Zhongnan Hospital of Wuhan University ethics committee (2021050K). Waiver of informed consent was approved for the aggregated data.

### Treatment

For patients who developed resistance to first-generation EGFR-TKI and had a secondary T790M mutation, the third-generation EGFR-TKI, osimertinib, would be preferred, while patients who were resistant to first-generation EGFR-TKI but do not have secondary T790M mutations, or those who have been resistant to both first- and third-generation EGFR-TKI, would be treated with chemotherapy or chemotherapy combined with ICI. The chemotherapy regimen after EGFR-TKI resistance (first- or third generation) was pemetrexed (500 mg/m^2^, Q3 weeks) in combination with cisplatin (75 mg/m^2^, Q3 weeks) or carboplatin (AUC 5), which was changed to pemetrexed (500 mg/m^2^, Q3 weeks) monotherapy after 4 cycles of doublet chemotherapy (intravenously).

Treatment options for patients receiving ICI-based therapies included ICI monotherapy or a combination of ICI with chemotherapy. ICI monotherapy was administered to patients with PS score >1 or those who were intolerant to chemotherapy. The chemotherapy regimens for combined ICIs were pemetrexed (500 mg/m^2^, Q3 weeks) plus cisplatin (75 mg/m^2^, Q3 weeks) or carboplatin (AUC 5). Patients receiving ICI in combination with chemotherapy would enter ICI maintenance therapy after 4 cycles of combined treatments. The details of ICI-based therapy are shown in **Supplementary Table S1**.

### Evaluation of treatment response and outcome

The mutation status of EGFR in all patients was detected by the next-generation sequencing technology (NGS) based on tumor biopsy specimens. Patients with atypical EGFR mutations were defined as those who harbored concomitant mutations or uncommon EGFR mutations. Treatment response was defined according to the Response Evaluation Criteria in Solid Tumors (RECIST) v.1.1. Overall survival (OS) was calculated from the date of immunotherapy or chemotherapy to the date of death from any cause or the date of final follow-up. Progression-free survival (PFS) was defined as the period from the date of immunotherapy or chemotherapy initiation to the date of disease progression or death from any cause or final follow-up.

Patients would undergo a comprehensive review after every two cycles of therapy, including imaging evaluations and laboratory tests, such as blood count, biochemical analyses (coagulation profile, and hepatic and renal function), thyroid function, and tumor marker tests.

### Statistical analysis

OS and PFS were evaluated using the Kaplan–Meier method. The log-rank statistic is approximately distributed as a chi-square test statistic with degree of freedom corresponding to the number of comparison groups minus 1. Multivariate Cox proportional hazard analysis was performed to determine the association of different covariates with OS and PFS. All analyses were carried out using SPSS Statistics, version 20.0 (IBM Corp., Armonk, NY). Statistical significance was at *P* ≤ 0.05. The *P* values were derived from a two-tailed test.

## Results

### Patient characteristics in the study cohort

From September 2018 to July 2020, a total of 132 metastatic NSCLC patients who developed EGFR-TKI resistance were included in our study. Their median age was 57 years (interquartile range [IQR], 52–64 years). In terms of treatment modality, 54.5% of patients received ICI-based therapy compared to 45.5% of patients who received chemotherapy alone. Those who received chemotherapy alone were not subsequently treated with ICI because of the accessibility of the medication and their disease. Their median number of treatment cycles was similar, at 6 and 7 cycles, respectively. The ICI-based treatment group showed a longer duration (≥12 months) of EGFR-TKI treatment and a higher proportion of T790M mutations as compared to the chemotherapy group. Their baseline characteristics are presented in **Table 1**.

**Table 1:** Baseline characteristics of the study cohort.

Characteristic	Patients N. (%)		*P*
	ICI-based therapy N = 72 (54.5)	chemotherapy N = 60 (45.5)	
Age group, years			
<65	58 (80.6)	47 (78.3)	0.75
≥65	14 (19.4)	13 (21.7)	
Sex			
Male	28 (38.9)	32 (53.3)	0.97
Female	44 (61.1)	28 (46.7)	
Smoking			
Yes	25 (34.7)	26 (43.3)	0.32
No	47 (65.3)	34 (56.7)	
Pathological type			
Adenocarcinoma	70 (97.2)	57 (95)	0.84
Squamous cell carcinoma	2 (2.8)	3 (5)	
Brain metastases initially			
Yes	10 (13.9)	13 (21.7)	0.24
No	62 (86.1)	47 (78.3)	
Liver metastases initially			
Yes	11 (15.3)	4 (6.7)	0.12
No	61 (84.7)	56 (93.3)	
EGFR mutation status at first biopsy			
Ex19Del alone	35 (48.6)	27 (45)	0.64
L858R alone	27 (37.5)	21 (35)	
Atypical EGFR mutations*	10 (13.9)	12 (20)	
First EGFR-TKI			
Gefitinib	56 (77.8)	41 (68.3)	0.43
Erlotinib	7 (9.7)	7 (11.7)	
Icotinib	9 (12.5)	12 (20)	
Pathological type at re-biopsy			
Adenocarcinoma Lowly differentiated cancer Unknown	31 (43.1) 6 (8.3) 35 (48.6)	24 (40.0) 6 (10.0) 30 (50.0)	0.91
EGFR mutation status at re-biopsy			
T 790M positive alone T790M positive combined with Ex19Del or L858R	15 (20.8) 16 (22.2)	11 (18.3) 10 (16.7)	0.71
T790M negative Unknown	27 (37.5) 14 (19.4)	23 (38.3) 16 (26.7)	
Osimertinib	28	15	0.09
Duration of EGFR-TKI therapy (months)			
<12	25 (26.4)	29 (48.3)	0.11
≥12	47 (73.6)	31 (51.7)	
Radiotherapy history			
Yes No	56 (77.8) 16 (12.2)	50 (83.3) 10 (16.7)	0.42
ECOG score			
1	57 (79.2)	51 (85.0)	0.39
2	15 (20.8)	9 (15.0)	
Therapy cycles (IQR)	6 (5 to 12)	7 (6 to 10)	
PD-L1 expression			
<1% 1%-49% ≥50% Unknown	22 (30.6) 12 (16.7) 3 (4.1) 35 (48.6)	15 (25.0) 10 (16.7) 5 (8.3) 30 (50.0)	0.73

IQR, interquartile range; ECOG, Eastern Cooperative Oncology Group; CNS, central nervous system; ICI, immune checkpoint inhibitor; EGFR, epidermal growth factor receptor.

*Including patients with concomitant mutations and/or uncommon EGFR mutations.

### Outcomes

**Figure 1:**
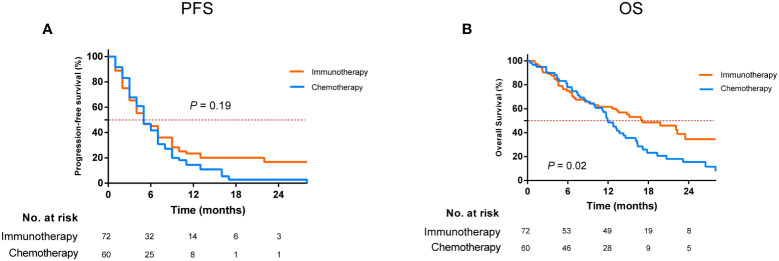
Comparison of progression-free survival **(A)** and overall survival **(B)** for patients who developed EGFR-TKI resistance treated with immunotherapy and chemotherapy alone. EGFR, epidermal growth factor receptor; TKI, tyrosine kinase inhibitor.

The median follow-up time was 21.7 months (IQR, 14.8–28.8 months) as of 11 October 2021. The median PFS of the study cohort was 4.9 months (IQR, 2.8–9.2 months), and the PFS at 1 year was 19.0% (95% confidence interval [CI], 12.6–26.3) (**Supplementary Figure S1A**). Univariate analysis showed that ICI-based therapy has a similar PFS in comparison to chemotherapy alone (*P* = 0.19, **Figure 1A**). Multivariate analysis demonstrated that having good Eastern Cooperative Oncology Group (ECOG) scores and absence of brain metastases contained a lower risk of progression; however, ICI-based therapy was not significantly linked to progression improvement (HR, 0.75; 95% CI, 0.49–1.13; *P* = 0.17) (**Table 2**).

**Table 2:** Multivariable analysis of PFS and OS in patients who received ICI-based therapy and chemotherapy after developing EGFR-TKI resistance.

Variable	PFS			OS		
	HR	95% CI	P value	HR	95% CI	P value
Sex						
Female Male	Reference 1.31	0.86-1.97	0.21	Reference 1.94	1.24-3.03	**0.00**
Age, years						
<65 ≥65	Reference 1.38	0.81-2.33	0.24	Reference 0.97	0.53-1.77	0.92
EGFR mutation status at first biopsy						
Atypical EGFR mutations L858R alone Ex19Del alone	Reference 1.25 0.95	0.69-2.28 0.51-1.78	0.47 0.88	Reference 0.94 0.89	0.49-1.82 0.44-1.79	0.86 0.74
Secondary T790M mutation						
Positive Negative vs.	Reference 0.85	0.56-1.30	0.45	Reference0.93	0.57-1.53	0.78
Duration of EGFR-TKI therapy, months						
≥12 <12	Reference 0.79	0.50-1.23	0.30	Reference 0.75	0.45-1.27	0.27
ECOG PS						
>1 1	Reference 0.32	0.18-0.56	**0.00**	Reference 0.34	0.19-0.63	**0.00**
Brain metastases						
Yes No	Reference 0.66	0.45-0.98	**0.04**	Reference 0.52	0.33-0.81	**0.00**
Liver metastases						
Yes No	Reference 0.67	0.38-1.18	0.17	Reference 0.58	0.31-1.08	0.09
Modality						
CT ICI-based therapy	Reference 0.75	0.49-1.13	0.17	0.55	0.34-0.88	0.01

PFS, progression-free survival; OS, overall survival; EGFR, epidermal growth factor receptor; HR, hazard ratio; ECOG, Eastern Cooperative Oncology Group; CT, chemotherapy.

*Including patients harboring concomitant mutations or uncommon EGFR mutations.

The bolded numbers represent the results at P<0.05.

The median OS was 13.5 months (IQR, 6.6–26.5 months), with 1- and 2-year OS of 55.4% (95% CI, 46.4%–63.6%) and 25.8% (95% CI, 16.9%–35.5%), respectively (**Supplementary Figure S1B**). ICI-based therapy could show a significant OS advantage over chemotherapy alone, which could achieve a median OS of 17.1 and 12.0 months, respectively (*P* = 0.02, **Figure 1B**). Multivariate analysis confirmed that ICI-based therapy was an independent contributor for improving OS (HR, 0.55; 95% CI, 0.34–0.88; *P* = 0.01). Meanwhile, female, having a good ECOG PS scores, and without brain metastases were also independent predictors for harboring the better OS (**Table 2**).

### The optimal modality for immunotherapy

**Figure 2:**
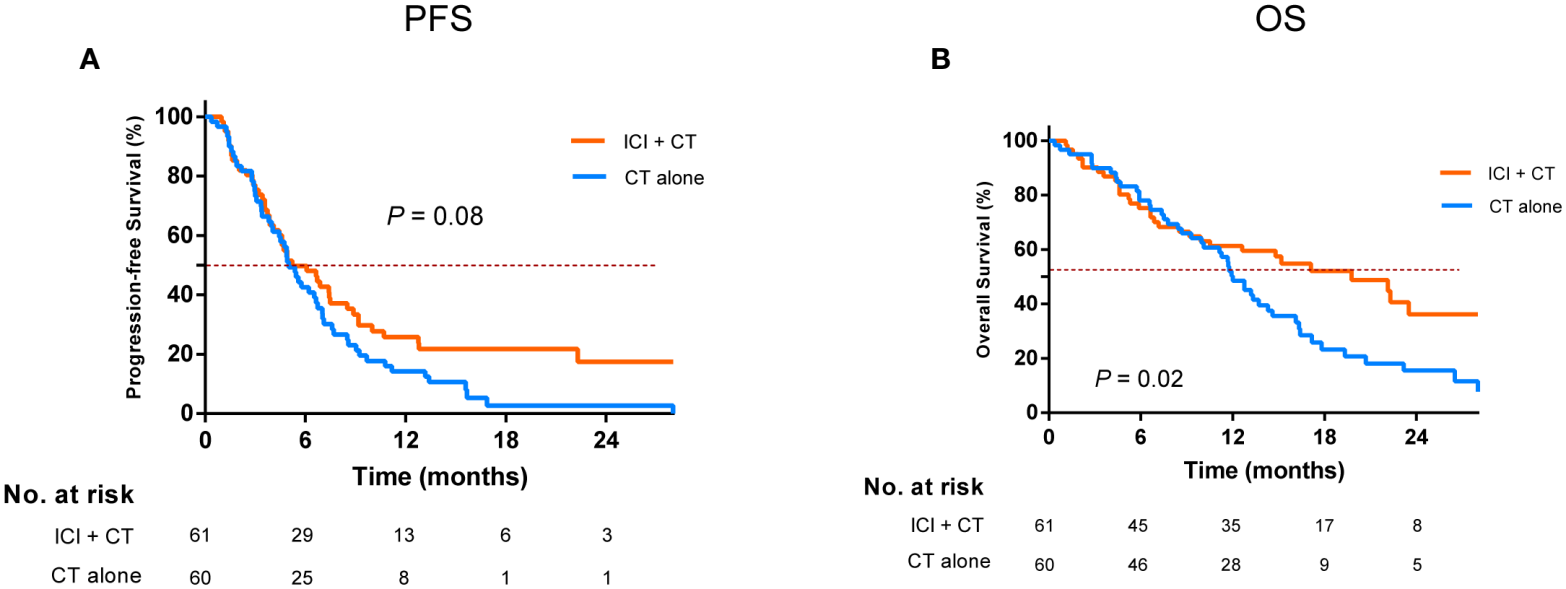
Comparison of progression-free survival **(A)** and overall survival **(B)** in the ICI combination chemotherapy versus the chemotherapy alone after EGFR-TKI resistance. EGFR, epidermal growth factor receptor; TKI, tyrosine kinase inhibitor; ICI, immune checkpoint inhibitor; CT, chemotherapy.

To determine the optimal mode of ICI-based therapy, we then performed a comparison of different treatment subgroups. We found that for EGFR-TKI-resistant patients, ICI plus chemotherapy resulted in the maximum improvement in OS relative to chemotherapy alone, yielding a corresponding median OS of 19.7 and 12.0 months, respectively (*P* = 0.02, **Figure 2B**); however, it only slightly prolonged median PFS from 5.0 to 5.2 months (*P* = 0.08, **Figure 2A**).

Furthermore, we concluded that patients who were younger (<65 years), have no T790M secondary mutations, or have good ECOG PS scores and those without brain or liver metastases were all the beneficiaries of the ICI-chemotherapy combination modality (**Table 3**).

**Table 3:** Subgroup analysis of the outcome of patients receiving ICI in combination with chemotherapy and chemotherapy alone.

Subgroups	ICI + CT	CT	HR for relapse(95% CI)	ICI + CT	CT	P value	HR for death(95% CI)	ICI + CT	CT	P value
	No. of patients			1-year PFS%				2-year OS%		
Overall	61	60	0.71 (0.48-1.05)	25.7	14.2	0.08	0.58 (0.37-0.91)	36.2	15.5	0.02
≥65 years	12	13	1.02 (0.43-2.45)	15.0	23.1	0.96	0.96 (0.34-2.74)	0.0	19.2	0.94
<65 years	49	47	0.69 (0.44-1.07)	27.4	11.6	0.09	0.55 (0.33-0.91)	39.2	14.3	0.02
T790M positive	28	22	0.56 (0.30-1.67)	33.6	9.6	0.07	0.66 (0.32-1.35)	35.9	21.2	0.29
T790M negative	33	38	0.88 (0.53-1.45)	19.8	16.7	0.60	0.54 (0.29-0.98)	36.4	10.2	0.04
ECOG PS =1	49	51	0.63 (0.40-0.98)	31.1	14.7	0.04	0.50 (0.30-0.84)	40.7	16.2	0.01
ECOG PS >1	12	9	1.05 (0.40-2.74)	0.00	11.1	0.92	0.81 (0.29-2.26)	0.0	11.1	0.69
Brain metastasis	20	23	0.58 (0.30-1.14)	27.1	0.00	0.11	0.73 (0.37-1.44)	16.7	15.7	0.35
No brain metastasis	41	37	0.83 (0.51-1.37)	24.7	23.7	0.47	0.50 (0.27-0.93)	50.6	16.4	0.03
Liver metastasis	18	7	0.52 (0.20-1.39)	7.5	0.0	0.19	0.36 (0.13-1.03)	14.0	0.0	0.05
No liver metastasis	43	53	0.62 (0.40-0.98)	32.4	15.8	0.04	0.48 (0.27-0.83)	46.2	17.3	0.01

ICI, immune checkpoint inhibitor; CT, chemotherapy; HR, hazard ratio; ECOG, PFS, progression-free survival; OS, overall survival; ECOG, Eastern Cooperative Oncology Group.

### Toxicities

The ICI-based treatment had similar treatment-related toxicities compared to chemotherapy alone, the most common of which included neutropenia, anemia, and fatigue, with incidences of 58.3% (N = 42) versus 61.7% (N = 37), 48.6% (N = 35) versus 65.0% (N = 39), and 19.4% (N = 14) versus 25% (N = 15), respectively.

Grade 3 or higher toxicities occurred mainly in chemotherapy-containing regimens (ICI plus chemotherapy or chemotherapy alone), with neutropenia being the most common at 7.4% (N = 9), followed by thrombocytopenia at 9.1% (N = 11). For those treated with ICI monotherapy, no grade 3 or higher toxicities were observed.

A patient developed G3 dermatitis after receiving two cycles of ICI plus chemotherapy. After discontinuation and symptomatic management, the severity of the dermatitis returned to G1.

## Discussion

To date, ICI-based therapy is regarded as a promising second-line option for metastatic NSCLC with EGFR-TKI resistance, but the optimal modality is still under investigation. Our investigation has shown that ICI-based therapy is a superior treatment to conventional chemotherapy, and ICI combined with chemotherapy should be the recommended treatment for those with good ECOG PS scores, without secondary T790M mutations, and without initial brain metastases or liver metastases.

Previous studies have confirmed a lack of response to ICIs in metastatic NSCLC patients with EGFR mutations, and one of the potential mechanisms could be the low expression of PD-L1 or absence of infiltrating T cells in the TME (6, 7, 15). However, as tumors continue to evolve, the TME may change accordingly and, therefore, EGFR-TKI resistance might enhance the response to ICIs (8, 9, 13). As reported from the EGFR^+^/ALK^+^ cohort in the ATLANTIC study, the use of durvalumab monotherapy could provide a favorable outcome in EGFR^+^/ALK^+^ patients, with a median PFS and OS of 1.9 and 13.3 months, respectively, if PD-L1 expression is greater than 25% (16, 17). In the present study, patients receiving immunotherapy achieved PFS and OS of 4.9 and 17.1 months, respectively, which seemed to be superior to those reported from ATLANTIC, even when PD-L1 expression could not be clarified. In this cohort, more than 90% of ICI modalities were ICI combined with chemotherapy, which would be a possible reason for the better prognosis.

Previous studies have shown that the combination of cytotoxic chemotherapy agents and immunotherapy could increase the possibility of *de novo* antigen cross-presentation in tumor tissues (18), downregulate the expression of immunosuppressive cells (19), enhance the infiltration of effector T cells (20), and ultimately, improve the response to immunotherapy (4, 21–23). Notably, an important recent single-arm phase II study showed that in EGFR-TKI-resistant NSCLC, the regime of ICI combined with chemotherapy could result in a favorable objective remission rate (ORR, 50%), PFS (7 months), and OS (23.5 months) (13). A retrospective study has also identified the value of ICI combination chemotherapy in metastatic NSCLC after the advent of EGFR-TKI resistance (24). In our study, ICI plus chemotherapy resulted in a PFS of 5.2 months and an OS of 19.7 months, respectively. Our data and the results of that prospective study may suggest that even in EGFR-TKI-resistant populations, the combination of chemotherapy and ICI could provide a good treatment response.

Previous literature has reported that chemotherapy alone could be the best modality when resistance to EGFR-TKI occurred (25). In this study, we compared head-to-head the outcomes of ICI-based therapy and chemotherapy alone and confirmed a significant prognostic advantage of ICI-based therapy, which was mainly reflected in the OS benefit (26, 27).

The lack of sufficient tissue samples for exploratory analysis is a limitation of this study. Therefore, we were only able to test a limited number of specimens for PD-L1 status prior to ICI treatment, and the results showed no significant difference in the proportion of patients with positive expression between the two groups. Therefore, further studies are needed to confirm whether PD-L1 status could predict the superiority of later-line ICI over chemotherapy. In addition, the heterogeneity of the immunotherapy regimens is also a shortcoming of this study. A series of published studies had shown that the ICI regimens in this study had a similar efficacy in NSCLC (23, 28, 29). Therefore, these inconsistent regimens might not significantly affect our outcomes.

## Conclusion

ICI-based therapy is a promising option for NSCLC developing EGFR-TKI resistance. For those with good ECOG PS scores, no secondary T790M mutations, and without initial brain metastases or liver metastases, ICI combined with chemotherapy should be the optimal modality.

## Data availability statement

The raw data supporting the conclusions of this article will be made available by the authors, without undue reservation.

## Ethics statement

The studies involving human participants were reviewed and approved by Zhongnan Hospital of Wuhan University ethics committee. Written informed consent for participation was not required for this study in accordance with the national legislation and the institutional requirements.

## Author contributions

YC and JY: data collection, conception and writing of the manuscript; OW: conception and review of the manuscript; BY: data collection and review of the manuscript; CJ, WZ, and GC: review of the manuscript; CX and JZ: advice and review of the manuscript. All authors contributed to the article and approved the submitted version.

## Funding

This study was funded by the National Natural Science Foundation of China (grant no. 81972852) and Health Commission of Hubei Province Scientific Research Project (grant no. WJ2021F108).

## Acknowledgments

We thank Dr. Yong Yang (Fujian Union Hospital) for providing advice on study design.

## Conflict of interest

The authors declare that the research was conducted in the absence of any commercial or financial relationships that could be construed as a potential conflict of interest.

## Publisher’s note

All claims expressed in this article are solely those of the authors and do not necessarily represent those of their affiliated organizations, or those of the publisher, the editors and the reviewers. Any product that may be evaluated in this article, or claim that may be made by its manufacturer, is not guaranteed or endorsed by the publisher.

## Abbreviations

NSCLC, non-small cell lung cancer; EGFR-TKI, epidermal growth factor receptor tyrosine kinase inhibitors; ICI, immune checkpoint inhibitor; ECOG PS, Eastern Cooperative Oncology Group performance scores.
