# Supplementary material for: Is ICI-based therapy better than chemotherapy for metastatic NSCLC patients who develop EGFR-TKI resistance? A real-world investigation
Source: Front Oncol. 2022 Aug 23;12:920047. doi: 10.3389/fonc.2022.920047 (PMC9445807; doi:10.3389/fonc.2022.920047)
Supplement: Supplementary file 1 [file DataSheet_1.docx]

**Supplementary appendix**

p 2. Supplementary table

p 2. Table S1. Immunotherapy regimes and dosage.

p 3. Supplementary figures

p 3. Figure S1. Progression-free survival and overall survival of patients who developed EGFR-TKI resistance.

**Table S1:** ICI-based regimes and dosage.

| **Regimes** | **Cases** | **Usage** |
| --- | --- | --- |
| **ICI monotherapy** |  |  |
| Pembrolizumab | 11 | 200 mg, iv. q3w |
|  |  |  |
| **Chemotherapy in combination with ICI*** |  |  |
| Pemetrexed + cisplatin / carboplatin in combination with pembrolizumab | 19 | Pemetrexed [500 mg/m^2^], cisplatin [75 mg/m^2^], carboplatin [AUC5], Pembrolizumab [200 mg], iv. q3w |
| Pemetrexed + cisplatin / carboplatin in combination with sintilimab | 25 | Pemetrexed [500 mg/m^2^], cisplatin [75 mg/m^2^], carboplatin [AUC5], Sintilimab [200 mg], iv. q3w |
| Pemetrexed + cisplatin / carboplatin in combination with toripalimab | 8 | Pemetrexed [500 mg/m^2^], cisplatin [75 mg/m^2^], carboplatin [AUC5], Toripalimab [240 mg] iv. q3w |
| Paclitaxel / docetaxel + cisplatin / carboplatin in combination with pembrolizumab | 9 | Paclitaxel [260 mg/m^2^] or docetaxel, [75 mg/m^2^], cisplatin [75 mg/m^2^], carboplatin [AUC5], Pembrolizumab [200 mg], iv. q3w |

Abbreviations: ICI, immune checkpoint inhibitor.


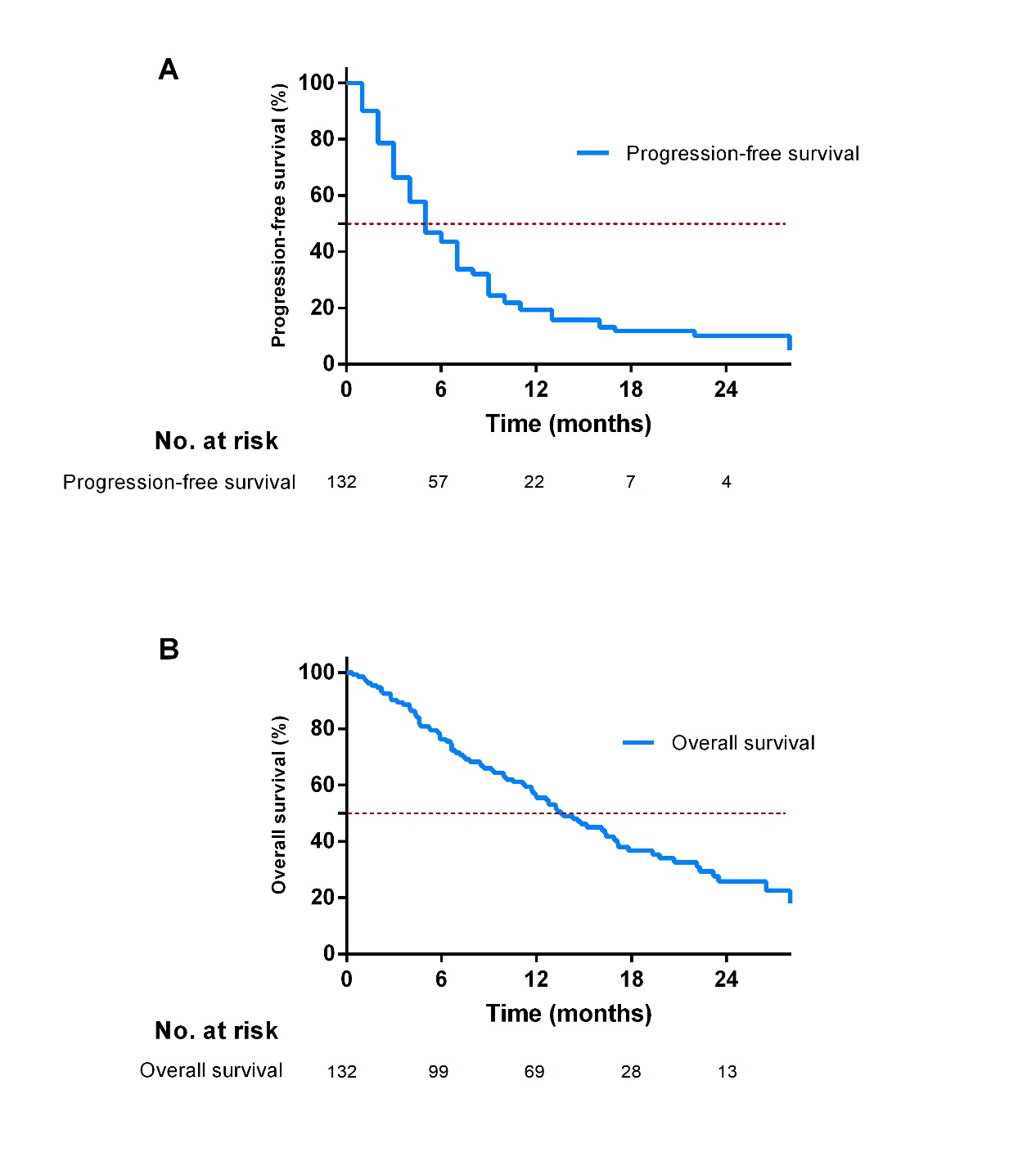


**Figure S1.** Progression-free survival (A) and overall survival (B) of the patients who developed the EGFR-TKI resistance. EGFR-TKI, epidermal growth factor receptor tyrosine kinase inhibitors.
